# Physicochemical and Digestible Properties of Parboiled Black Rice With Different Amylose Contents

**DOI:** 10.3389/fnut.2022.934209

**Published:** 2022-07-07

**Authors:** Wei Zhang, Bei Cheng, Xuefeng Zeng, Qiuling Tang, Zaixi Shu, Pingping Wang

**Affiliations:** ^1^College of Food Science and Engineering, Wuhan Polytechnic University, Wuhan, China; ^2^Key Laboratory for Deep Processing of Major Grain and Oil (Wuhan Polytechnic University), Ministry of Education, Wuhan, China; ^3^School of Liquor and Food Engineering, Guizhou University, Guiyang, China

**Keywords:** black rice, parboiling, crystalline structure, cooking, digestibility

## Abstract

The varieties of black rice with different amylose contents (waxy; medium-amylose: 16.03%; high-amylose: 27.14%) were parboiled and then evaluated for physicochemical and digestible properties. The color, crystalline structure, and swelling property of parboiled rice were analyzed, and the water molecular mobility, texture, and starch digestibility of cooked parboiled rice were determined. The color of black rice was only slightly changed after the parboiling treatment. The crystalline structures of waxy and medium-amylose rice were severely damaged by the parboiling treatment, while the highly crystalline structure was retained in parboiled high-amylose rice. During heating in water, parboiled high-amylose rice had a lower water absorption ratio (WAR) and volume expansion ratio (VER) than the other two varieties. After cooking, parboiled high-amylose rice had higher water molecular mobility and harder texture compared with the other two varieties. Cooked parboiled high-amylose rice contained higher content of resistant starch than cooked parboiled waxy and medium-amylose rice.

## Introduction

Rice (*Oryza sativa* L.) is one of the most significant cereals in the world and serves as a staple food source for more than 3 billion people. Black rice is rare rice germplasm that has various health benefits (1). The dark color of black rice is attributed to the high anthocyanin content, located in the pericarp layers. The anthocyanins of black rice can ameliorate insulin resistance (2), alleviate hyperuricemia (3), improve gut microbiota dysbiosis (4), and inhibit the growth of cancer cells (5).

Parboiling is a hydrothermal treatment applied to rough rice grains, which includes three main procedures: soaking, steaming, and drying (6). The parboiling treatment can improve the oxidative stability of rice and prolong its storage (7). Parboiling can increase the bioavailability of minerals in rice, as minerals seep into the endosperm from the bran layer (8). During the parboiling treatment, starch undergoes gradual gelatinization, which can bring about changes in the physicochemical properties of rice. Parboiling can enhance the compactness of rice kernels and reduce starch digestibility (9).

Amylose content is the most crucial factor for determining the structure and properties of rice starch, thereby affecting the qualities of rice and rice products. Teng et al. (10) reported that amylose content had a reverse correlation with the relative crystallinity and swelling power of rice. Govindaraju et al. (11) suggested that amylose content tends to reversely affect the gelatinization temperature of rice starch. Teixeria et al. (12) found that white rice which contained a lower proportion of amylose had higher starch digestibility. Dutt et al. (13) indicated that there was a positive correlation between amylose content and hardness of cooked rice.

In many Asian and European countries, parboiled rice is more accepted than milled rice. In recent years, the regulable digestibility of parboiled rice has gained a growing interest of manufacturers and food scientists. However, to date, the structure and properties of parboiled black rice have not been well-understood. This research aimed to interpret the effect of amylose content on the physicochemical properties and digestibility of parboiled black rice. This research meets the expanding demand of humans for healthy diets and can also promote the development of the black rice industry.

## Materials and Methods

### Materials

Waxy, medium-amylose, and high-amylose black rice (rough rice, varieties of Xuenuomi, Heizhenzhu, and Haishuidao) were supplied by a grain trader (Hubei, China). Total starch assay kit and glucose oxidase-peroxidase (GOPOD) reagent were obtained from Megazyme International Ireland Ltd. (Bray Co., Wicklow, Ireland). Pancreatic α-amylase (P7545), pepsin (P7000), and amyloglucosidase (A7095) were purchased from Sigma Chemical Co. (St. Louis, MO., U.S.A.). All other chemicals were of analytical grade unless otherwise stated.

### Proximate Analysis

Rough black rice was dehulled with a Rice huller (JLGJ 4.5, Taizhou Grain Meter Factory, Taizhou, China). The total starch content of brown rice was determined with the total starch assay kit according to American Association of Cereal Chemists International (AACCI) Approved Method 76-13.01 (14). The amylose content of rice was measured by the method described by Lin et al. (15). The protein content of rice was determined using Association of Official Analytical Chemistry (AOAC) Official Method 992.23 (16). The fat content of rice was measured following the AACCI Approved Method 30-20.01 (17).

### Pasting Property Measurement

Pasting properties of rice flours were analyzed using a Newport Scientific rapid visco-analyzer (RVA) (Warriewood, New South Wales, Australia) according to the method of Liu et al. (18) with minor modifications. Flour suspensions (12% w/w) were held at 50°C for 1 min, heated to 95°C at a rate of 12°C/min, maintained at 95°C for 2.5 min, cooled to 50°C at a rate of 12°C/min, and finally held at 50°C for 1.5 min. The rotation speed of the paddle was set at 960 rpm for the initial 10 s and then 160 rpm for the remaining test.

### Preparation of Parboiled Black Rice

Parboiled blacked rice samples were prepared according to the method of Leethanapanich et al. (19) with minor modifications. One hundred grams (dry weight) of rough black rice was put into boiled water (150 ml). When the temperature of the water was cooled down to 65°C, the temperature was kept constant for soaking for 4 h. The soaked rice was steamed for 20 min using an MG-TH559 rice cooker (Foshan Shunde Median Home Appliance Co. Ltd., Foshan, Guangdong, China). The steamed rice was firstly dried at 70°C for 1 h, naturally cooled for 40 min, and then secondly dried at 65°C for 40 min. The dried rice was naturally cooled to room temperature overnight. Parboiled black rice was obtained by dehulling the dried steamed rice with the Rice huller (JLGJ 4.5, Taizhou, China). The parboiled rice was grounded to flour, which pass through 100-mesh screen. The flour was used for the analysis of X-ray diffraction (XRD) and starch digestibility.

### Color Measurement of Black Rice

The color of black rice was measured with a CR210 Chroma Meter (Minolta Corp., Osaka, Japan). A standard white tile was used to calibrate the instrument. In CIELAB color space, L^*^ describes lightness/darkness, a^*^ describes redness/greenness, and b^*^ describes yellowness/blueness.

### Determinations of Water Absorption Ratio and Volume Expansion Ratio of Parboiled Rice During Heating in Water

The samples of parboiled rice (5 g) were heated in different temperatures (80, 90, and 100 °C) of water (30 ml) for different durations (15, 30, and 45 min). After the removal of surface water with filter paper, heated samples were weighed. An increase in parboiled rice weight was calculated. The WAR of parboiled rice was expressed as the ratio of heated rice to that of the unheated sample (20). The volumes of both heated and unheated parboiled rice were measured. The VER of parboiled rice was expressed as the ratio of the volume of the heated sample to that of the unheated sample (21).

### X-Ray Diffraction Analysis

An X-ray diffractometer (PANalytical B.V., Almelo, The Netherlands) with Cu radiation was used to analyze the crystalline structures of rice flours according to the method of Zhu et al. (22) with minor modifications. The operating voltage and current were 30 kV and 30 mA, respectively. All samples were scanned over an angular range from 3° to 40°, with a step size of 0.02.

### Cooking Process of Parboiled Rice

Parboiled rice (20 g) was soaked in distilled water (1.2 volume folds of rice) for 10 min before cooking. Parboiled rice was steamed for 30 min using the MG-TH559 rice cooker (Guangdong, China). Cooked parboiled rice was frozen using liquid nitrogen and then lyophilized. The dried sample was ground into flour, which pass through a 100-mesh screen. The resultant flour was used for the *in vitro* analysis of starch digestibility.

### Low-Field Nuclear Magnetic Resonance Measurement

Proton distributions of cooked rice samples were measured using an NMI20-040V-I LF-NMR analyzer (Niumai Instruments, Suzhou, China). Cooked parboiled rice grains were balanced at 25°C for 20 min before measurement. The transverse relaxation (T_2_) curves were obtained using the Carr-Purcell-Meiboom-Gill (CPMG) pulse sequence. The measurement parameters were as follows: receiver bandwidth = 200 kHz, repeat sampling wait time = 2,000 ms, analog gain = 15db, digital gain = 3, pre-amplifier gain = 3, number of echoes = 10,000, echo time = 0.1 ms, and scan number = 16.

### Texture Analysis

Texture parameters of the cooked parboiled rice were measured using a Texture Analyzer (TA.XT2i, Stable Micro System, Surrey, UK). A two-cycle compression test was performed with compression rate and compression speed set at 70% and 1 mm/min, respectively.

### *In vitro* Assay of Starch Digestibility

Starch digestibilities of uncooked and cooked parboiled rice were analyzed following the method described by Englyst et al. (23) with minor modifications. Pancreas α-amylose (3.6 g) was added to 32 ml of water under magnetic stirring. The dispersion was centrifuged at 10,000 × g for 10 min, and 21.6 ml of the supernatant was collected into a beaker. Amyloglucosidase (2.56 ml) was diluted to 3.2 ml, and 2.4 ml of the diluent was then added to the beaker containing α-amylose solution. The enzyme mixture was prepared freshly for the digestion assay.

One hundred milligrams (dry weight) of rice flour was added to centrifuge tubes. Ten milligrams of pepsin and 2 ml of HCl (0.025 mol·l^−1^) were added to each tube. Tubes were incubated in a 37°C water bath under agitation (150 rpm). After 30 min of incubation, each tube was added with 5 ml of sodium acetate buffer (0.5 mol·l^−1^, pH 5.2). Tubes were incubated in the water bath for another 5 min. Following this, the α-amylose/amyloglucosidase mixed solution (1 ml) was added to each tube, which was then further incubated in the water bath for digestion of starches. After 20 and 120 min of digestion, 0.1 ml of the hydrolyzate was collected and added with 4 ml of 95% ethanol to terminal the enzymatic reaction. The released glucose of the hydrolyzates was measured using GOPOD reagent, which was used to calculate the contents of rapidly digestible starch (RDS), slowly digestible starch (SDS), and resistant starch (RS).

### Statistical Analysis

Experiments were conducted in triplicate. Statistical analysis was done using SPSS 25 software (SPSS Inc., Chicago, IL, USA). The one-way analysis of variance and Duncan's test were performed to compare means at a significance level of 5%.

## Results and Discussion

### Chemical Composition of Raw Black Rice

The chemical compositions of raw black rice with different amylose contents are shown in Table 1. The amylose contents of medium-amylose and high-amylose black rice were 16.03 and 27.14%, respectively. Waxy and medium-amylose black rice contained higher content of starch but lower content of protein than the high-amylose variety. There was no significant difference in the starch content of waxy and medium-amylose black rice, but the protein content of waxy rice was slightly higher than that of the medium-amylose variety. The three varieties of rice only had a slight difference in fat content.

**Table 1:** Chemical compositions of raw black rice with different amylose contents^†^.

**Rice variety**	**Moisture (%)**	**Starch (%)**	**Amylose (%)**	**Protein (%)**	**Fat (%)**
Waxy	11.01 ± 0.40^a^	79.01 ± 0.45^b^	-	8.48 ± 0.05^b^	3.29 ± 0.01^c^
Medium-amylose	12.06 ± 0.64^b^	76.72 ± 0.99^b^	16.03 ± 0.04^a^	7.83 ± 0.03^a^	3.09 ± 0.01^a^
High-amylose	12.00 ± 0.72^b^	72.10 ± 1.35^a^	27.14 ± 0.33^b^	11.67 ± 0.15^c^	3.19 ± 0.02^b^

† *Data were chemical compositions of brown rice. Data were expressed as mean ± SE. Data with the same letter in the same column were not significantly different at a significance level of 0.05*.

### Pasting Properties of Raw Black Rice

**Figure 1:**
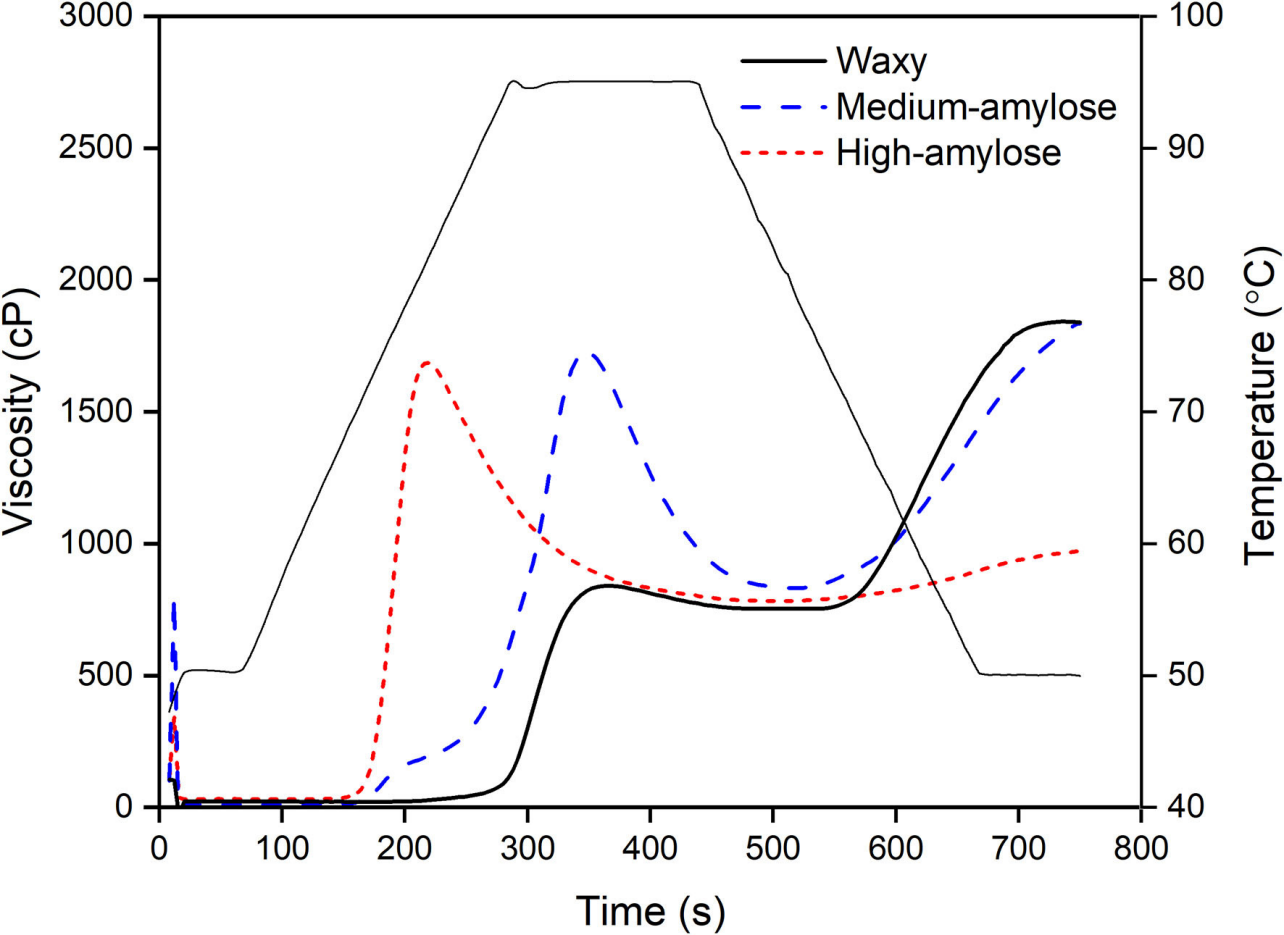
Pasting curves of waxy, medium-amylose, high-amylose black rice.

The pasting curves of the three varieties of raw black rice are shown in Figure 1, and their pasting parameters are shown in Table 2. The pasting temperature (PT) of black rice increased with increasing amylose content. Amylose can suppress the swelling of starch granules and maintain granular integrity during swelling (24). Li et al. (25) reported that PT of wheat flour was positively correlated with amylose content. Waxy black rice and medium-amylose black rice did not have significant differences in peak viscosity (PV) and breakdown viscosity (BDV) and only had a slight difference in trough viscosity (TRV). PV, TRV, and BDV of high-amylose black rice were significantly lower than those of the other two rice varieties. Gani et al. (26) reported that high-amylose starch attained the PV at a higher temperature than low-amylose starches, thus presenting lower PV. Arcangelis et al. (27) suggested that the lower BDV of high-amylose starch was attributed to the higher resistance of granules to shear force and disintegration. The final viscosities (FVs) and setback viscosities (SBVs) of medium- and high-amylose black rice were significantly higher than waxy rice. This result might be due to the different retrogradation rates of amylose and amylopectin. Retrogradation occurs faster for amylose than amylopectin, due to amylopectin's highly branched structure (28).

**Table 2:** Rapid visco-analyzer (RVA) parameters of raw black rice with different amylose contents^†^.

**Rice variety**	**PT (**°**C)**	**PV (cP)**	**TRV (cP)**	**BDV (cP)**	**FV (cP)**	**SBV (cP)**
Waxy	71.0 ± 0.4^a^	1,710 ± 24^b^	786 ± 5^b^	924 ± 20^b^	980 ± 5^a^	194 ± 5^a^
Medium-amylose	88.3 ± 0.9^b^	1,730 ± 4^b^	808 ± 24^c^	923 ± 28^b^	1,811 ± 24^b^	1,004 ± 1^b^
High-amylose	94.7 ± 0.1^c^	836 ± 5^a^	756 ± 3^a^	80 ± 8^a^	1,806 ± 33^b^	1,050 ± 36^c^

† *Data were expressed as means ± SD. Means within a column that had the same letter were not significantly different (α = 0.05). PT, pasting temperature; PV, peak viscosity; TRV, trough viscosity; BDV, breakdown viscosity; FV, final viscosity; SBV, setback viscosity*.

### Color of Parboiled Black Rice

Color values of raw and parboiled black rice are presented in Table 3. L^*^, a^*^, and b^*^ values of raw black rice were in the range of 55.13~57.43, −6.61~-1.49, and 1.33~5.00, respectively. There were slight differences among the three black rice varieties in color values of raw kernels. After parboiling treatment, all three black rice underwent a slight change in color values. Compared with raw rice, the L^*^ and a^*^ values of parboiled black rice slightly decreased, indicating a decrease in brightness and an increase in greenness. After parboiling treatment, the b^*^ value of waxy and medium-amylose rice slightly decreased, suggesting that the parboiling process decreased the yellowness of the two varieties of rice. The L^*^, a^*^, and b^*^ values of parboiled black rice were in the range of 54.63~56.27, −6.86~-4.53, and 1.40~2.81, respectively.

**Table 3:** Color of raw and parboiled black rice with different amylose contents^†^.

**Rice variety**	L*	a*	b*
**Raw rice**			
Waxy	57.43 ± 0.09^b^	−3.90 ± 0.09^b^	3.63 ± 0.04^b^
Medium-amylose	58.56 ± 0.05^c^	−1.49 ± 0.45^c^	5.00 ± 0.14^c^
High-amylose	55.13 ± 0.05^a^	−6.61 ± 0.05^a^	1.33 ± 0.05^a^
**Parboiled rice**			
Waxy	56.27 ± 0.12^b^	−5.01 ± 0.17^b^	2.73 ± 0.12^b^
Medium-amylose	56.23 ± 0.05^b^	−4.53 ± 0.21^c^	2.81 ± 0.08^b^
High-amylose	54.63 ± 0.05^a^	−6.86 ± 0.05^a^	1.40 ± 0.14^a^

† *Data were expressed as means ± SD. For raw rice or parboiled rice, means within a column that had the same letter were not significantly different (α = 0.05)*.

### Crystalline Structure of Parboiled Black Rice

**Figure 2:**
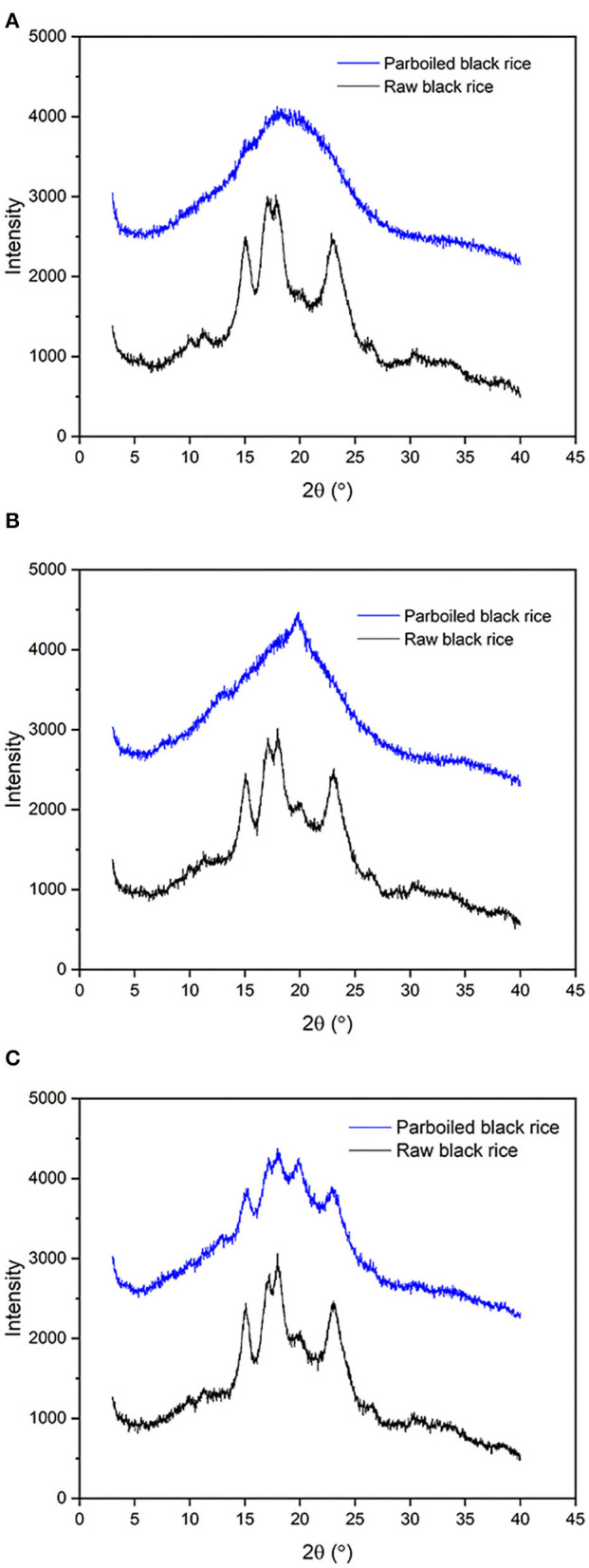
X-ray diffraction (XRD) patterns of raw and parboiled black rice. **(A)** waxy black rice; **(B)** medium-amylose black rice; **(C)** high-amylose black rice.

The XRD patterns of raw and parboiled black rice are shown in Figure 2. For raw rice, all the three varieties of black rice exhibited characteristic peaks at 2θ of 15.0°, 17.0°, 17.8°, and 23.0°, indicating an A-type crystalline structure of the included starch (29). After parboiling treatment, waxy black rice exhibited an amorphous pattern. This result suggested that the parboiling process completely destroyed the crystalline structure of starch in waxy rice. Medium-amylose rice lost most of the crystalline structure after parboiling treatment. For high-amylose black rice, the parboiling process greatly reduced the intensity of four characteristic peaks, but the parboiled product still retained a highly crystalline structure. These results suggested that the crystalline structure of parboiled black rice was related to the amylose content of the rice variety. As discussed above in the pasting property, elevating the amylose content could enhance the tightness of starch granules and restrain the swelling of granules upon heating.

### Water Absorption and Volume Expansion of Parboiled Rice During Heating in Water

**Figure 3:**
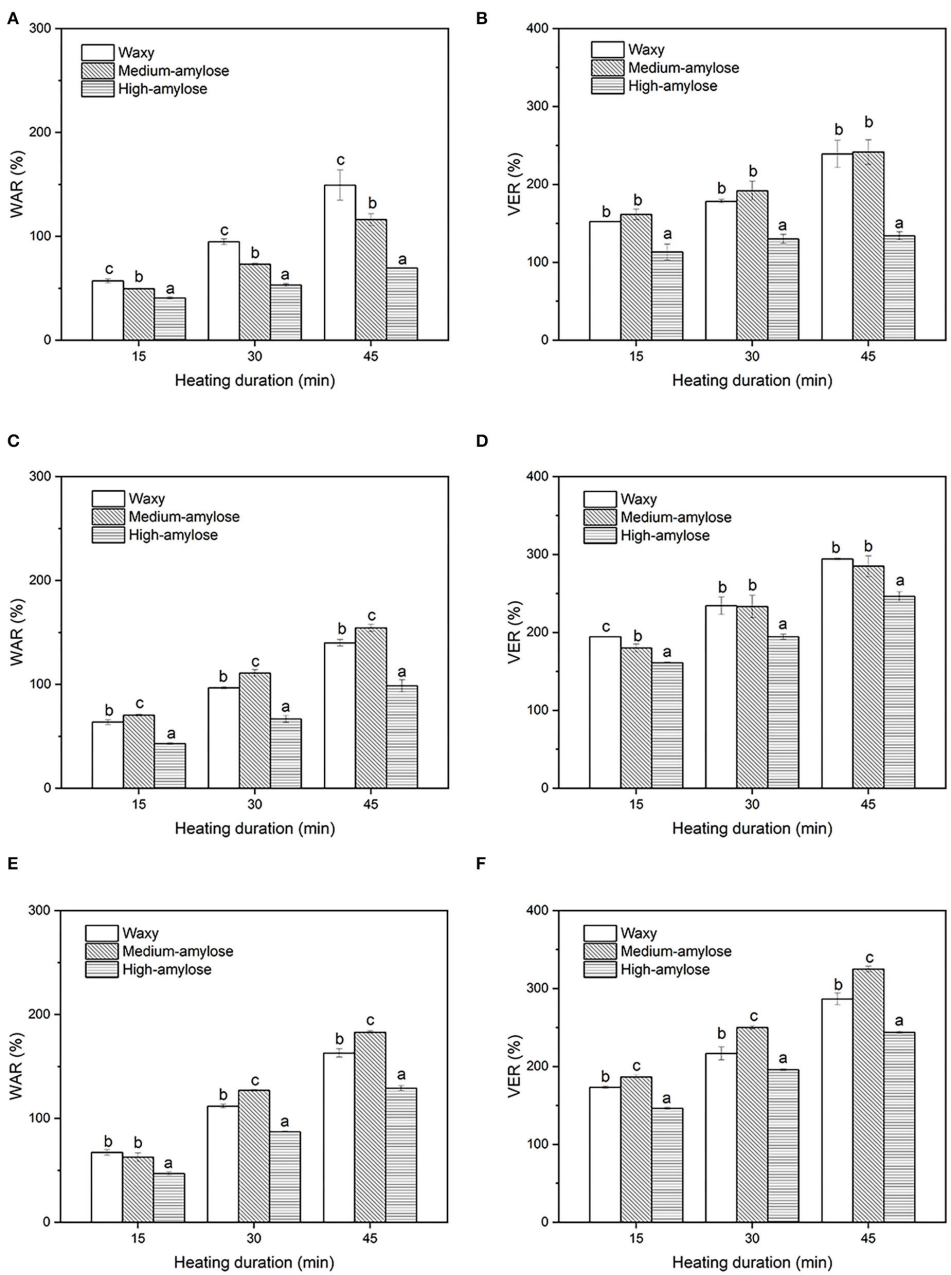
Water absorption ratio (WAR) and volume expansion ratio (VER) of parboiled black rice heated in different temperatures of water. **(A)** WAR-80°C; **(B)** VER-80 °C; **(C)** WAR-90 °C; **(D)** VER-90 °C; **(E)** WAR-100 °C; **(F)** VER-100 °C. For a specific heating duration in each subfigure, columns with the same letter were not significantly different (a = 0.05).

The WAR and VER of parboiled rice during heating at different temperatures are shown in Figure 3. At all three heating temperatures (80, 90, and 100 °C), the WAR and VER of parboiled rice increased with increasing heating duration. For the same heating treatment, parboiled high-amylose rice had lower WAR and VER than the other two varieties. There were slight differences between parboiled waxy and medium-amylose in WAR and VER. The low WAR and VER of parboiled high-amylose rice might be due to retained crystalline structure of starch after the parboiled process. The strong interaction of chains in the crystalline zone restrained the swelling of rice kernels. Sui et al. (30) reported that after heat moisture treatment, high-amylose rice starch had lower swelling power than waxy and medium-amylose rice starch.

### Water Molecular Mobility of Cooked Parboiled Black Rice

**Figure 4:**
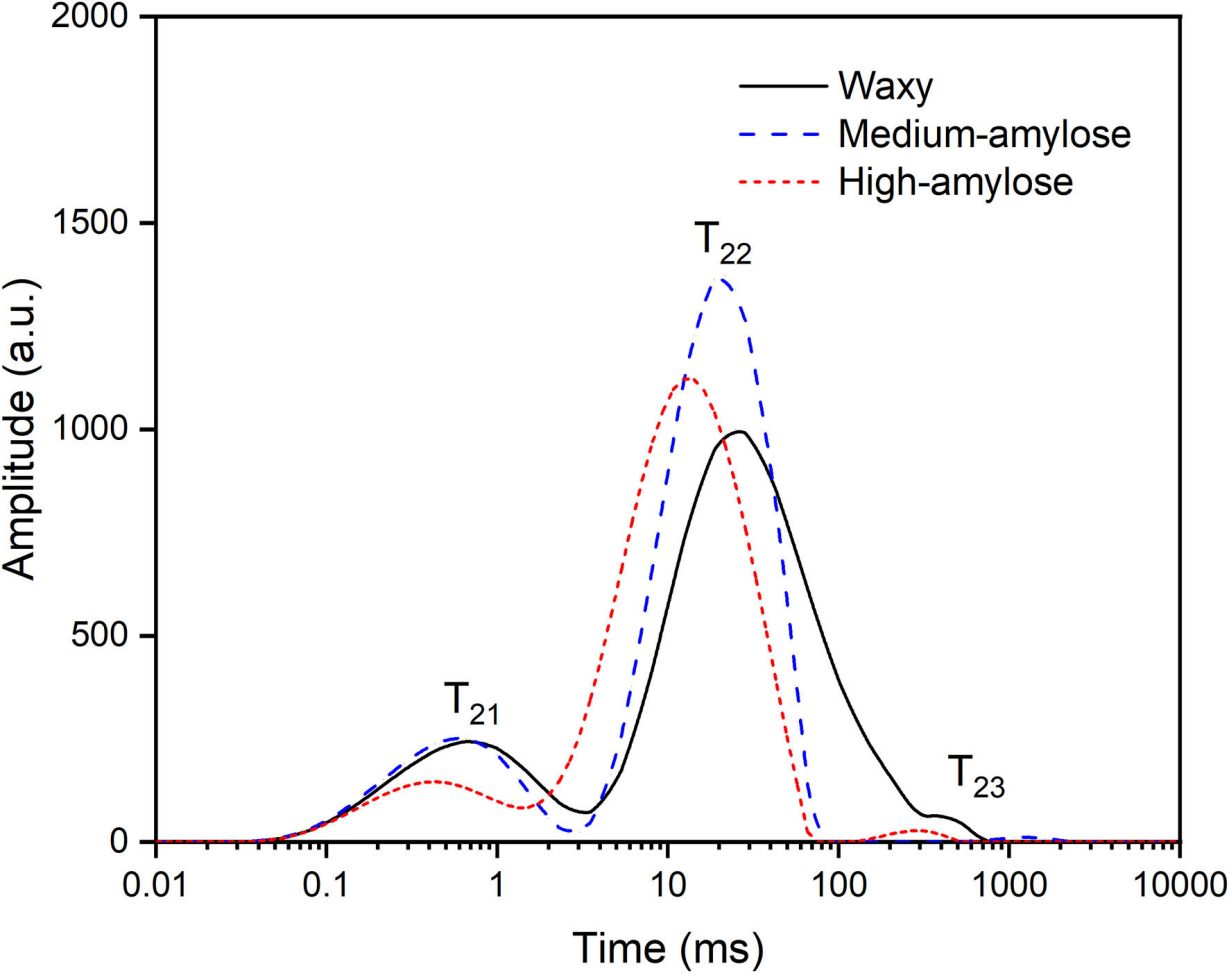
Proton distributions of cooked parboiled black rice.

**Figure 5:**
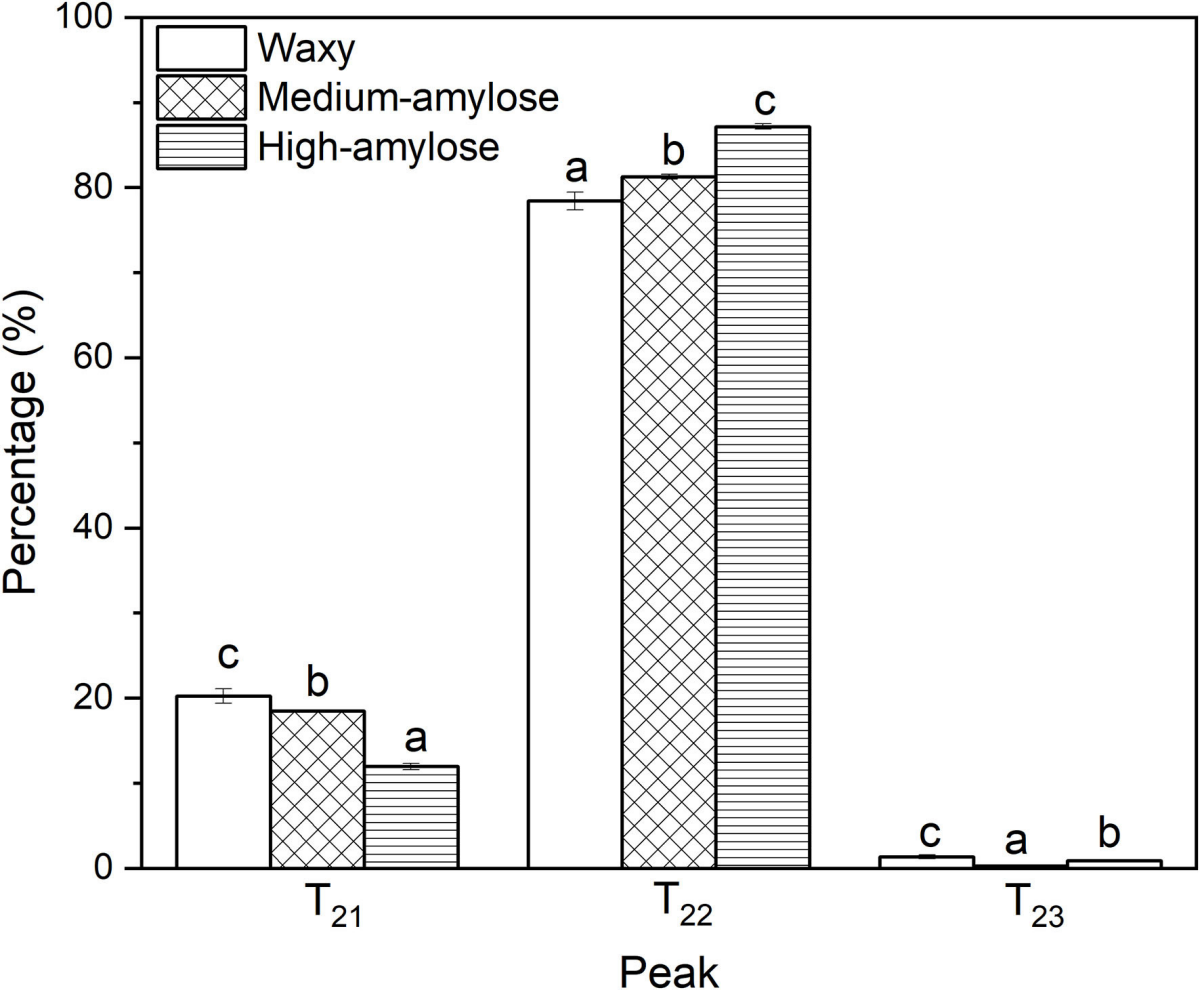
Peak percentages of T_21_ (tightly bound water), T_22_ (weakly bound water), and T_23_ (free water) in cooked parboiled black rice. For a specific peak, columns with the same letter were not significantly different (a = 0.05).

The proton distributions of cooked parboiled black rice with different amylose contents are shown in Figure 4. Peaks of -T_21_ (0.1–1 ms), T_22_ (1–100 ms), and T_23_ (100–1,000 ms) were assigned to tightly bound, weakly bound, and free water in the parboiled rice (31). Peak area percentages (A_21_, A_22_, and A_23_) of T_21_, T_22_, and T_23_, reflecting three kinds of water, are shown in Figure 5. For cooked parboiled rice of all three rice varieties, weakly bound water accounted for the largest proportion of the total water (78.4~87.2%), followed by tightly bound water (12.0~20.2%). This result was generally consistent with the previous research on cooked rice (32). The three varieties of cooked parboiled rice exhibited certain differences in proportions of tightly and weakly bound water. As amylose content increased, the tightly bound water of cooked parboiled rice decreased, but the weakly bound water increased. Amylose has a linear chain structure and is prone to reassemble after the cooking process. The interaction between amylose chains can increase the water molecular mobility of cooked parboiled rice.

### Texture Attributes of Cooked Parboiled Black Rice

The texture attributes of cooked parboiled black rice are presented in Table 4. The hardness, cohesiveness, and gumminess of cooked parboiled medium-amylose rice were slightly higher than those of cooked parboiled waxy rice. Compared with cooked products of parboiled waxy and medium-amylose rice, the cooked parboiled high-amylose exhibited a great increase in texture parameters. Amylose was more readily retrograde than amylopectin. Mariotti et al. (33) found that for the gel of rice flour, the elevation of amylose content can strengthen the resistance of gel to stress. Gayin et al. (34) reported that the hardness and springiness of cooked rice were positively correlated with amylose content. For parboiled high-amylose rice, the restrained starch swelling might also contribute to the hard texture of the cooked product (35).

**Table 4:** Texture attributes of cooked parboiled black rice with different amylose contents^†^.

**Rice variety**	**Hardness**	**Springiness**	**Cohesiveness**	**Gumminess**
Waxy	1,538.2 ± 46.8^a^	0.51 ± 0.02^a^	306.2 ± 37.7^a^	597.3 ± 74.0^a^
Medium-amylose	1,695.3 ± 132.9^b^	0.53 ± 0.03^a^	390.1 ± 62.7^b^	728.8 ± 110.5^b^
High-amylose	4,180.6 ± 8.4^c^	0.69 ± 0.05^b^	1,221.1 ± 137.3^c^	1,766.7 ± 79.5^c^

† *Data were expressed as means ± SD. Means within a column that had the same letter were not significantly different (α = 0.05)*.

### Starch Digestibility of Cooked Parboiled Black Rice

The RDS, SDS, and RS contents of uncooked and cooked parboiled black rice are presented in Table 5. The RDS, SDS, and RS contents of uncooked parboiled were in the ranges of 25.83–57.84, 30.43–45.84, and 8.22–28.32%, respectively. The RS and RDS contents of uncooked high-amylose parboiled rice were significantly higher than those of waxy and medium-amylose cultivars. The high RS content of uncooked parboiled high-amylose rice might be due to the high crystalline structure of starch after the parboiling process. The parboiling treatment greatly damaged the crystalline structure of starch in waxy and medium-amylose rice, while highly crystalline structure remained in the parboiled product of high-amylose rice. For starch, the existence of crystalline zones inhibited the access of hydrolytic enzymes (36). After cooking, all three parboiled rice exhibited an increase in RDS content and a decrease in RS content. The rice kernels absorbed water during the cooking process and the volume expansion of starch accelerated the enzymatic hydrolysis of starch molecules. For parboiled waxy and medium-amylose rice, a low level of RS was observed after cooking. However, the cooked parboiled high-amylose rice still contained a certain content of RS (13.23%). Zheng et al. (37) reported that the increase of amylose content restrained the swelling of starch gel during cooking, leading to the elevation of RS content.

**Table 5:** Rapidly digestible starch (RDS), slowly digestible starch (SDS), and resistant starch (RS) contents of starch in parboiled and cooked parboiled black rice^†^.

**Rice variety**	**RDS (%)**	**SDS (%)**	**RS (%)**
**Parboiled rice**			
Waxy	57.84 ± 1.14^c^	30.43 ± 1.94^a^	11.72 ± 3.08^b^
Medium-amylose	51.77 ± 0.81^b^	40.01 ± 1.19^b^	8.22 ± 0.34^a^
High-amylose	25.83 ± 1.09^a^	45.84 ± 1.24^c^	28.32 ± 0.16^c^
**Cooked parboiled rice**			
Waxy	60.31 ± 0.38^b^	38.42 ± 0.54^b^	1.26 ± 0.14^a^
Medium-amylose	61.48 ± 0.41^c^	36.35 ± 0.33^a^	2.17 ± 0.21^b^
High-amylose	43.14 ± 1.14^a^	43.63 ± 0.93^c^	13.23 ± 0.40^c^

† *Data were expressed as means ± SD. For uncooked or cooked parboiled rice, means within a row that had the same letter were not significantly different (α = 0.05)*.

## Conclusion

High-amylose black rice had higher PT and lower PV, TRV, and BDV than the other two rice varieties. The parboiling treatment only slightly changes the color of black rice. The parboiling process severely destroyed the crystalline structure of waxy and medium-amylose rice, but only partly damaged the crystalline structure of the high-amylose variety. During heating, parboiled high-amylose rice had lower WAR and VER than those of the other two varieties. After cooking, parboiled high-amylose rice had higher water molecular mobility than the other two varieties. The cooked parboiled high-amylose rice had a harder texture and higher RS content than cooked parboiled waxy and medium-amylose rice. These results can provide a reference for the design of black rice products for the managements of type 2 diabetes and obesity.

## Data Availability Statement

The original contributions presented in the study are included in the article/supplementary material, further inquiries can be directed to the corresponding author.

## Author Contributions

WZ: conceptualization, writing the original draft, and funding acquisition. BC: investigation. XZ and PW: methodology and validation. QT and ZS: visualization and writing, reviewing, and editing the manuscript. All authors contributed to the article and approved the submitted version.

## Funding

This research was kindly supported by the Open Fund of Key Laboratory for Deep Processing of Major Grain and Oil (Wuhan Polytechnic University), Ministry of Education [Grant Number 2020JYBQGDKFB09].

## Conflict of Interest

The authors declare that the research was conducted in the absence of any commercial or financial relationships that could be construed as a potential conflict of interest.

## Publisher's Note

All claims expressed in this article are solely those of the authors and do not necessarily represent those of their affiliated organizations, or those of the publisher, the editors and the reviewers. Any product that may be evaluated in this article, or claim that may be made by its manufacturer, is not guaranteed or endorsed by the publisher.
